# Adverse Childhood Experiences and Psychological Correlates in College Students: A Comparison of Student-Athletes and Non-Student-Athletes

**DOI:** 10.3390/sports13070194

**Published:** 2025-06-20

**Authors:** Matthew D. Powless, Zachary A. Pilot, Elisabeth R. Brown, Mikaila C. Ealum, Kaitlyn N. Back, Sabrina Yamashita, Kaitlin Mindiola

**Affiliations:** 1 Department of Psychology and Behavioral Sciences, University of Evansville, 1800 Lincoln Ave. Evansville, IN 47714, USA; sy64@evansville.edu (S.Y.); km617@evansville.edu (K.M.); 2 Psychology Department, University of Southern Indiana, 8600 University Blvd., Evansville, IN 47712, USA; zpilot@usi.edu (Z.A.P.); erbrown3@eagles.usi.edu (E.R.B.); katienback@gmail.com (K.N.B.); 3 Department of Counseling and Educational Psychology, Indiana University, 201 N. Rose Ave., Bloomington, IN 47405, USA; mcealum@iu.edu

**Keywords:** ACEs, student-athletes, depression, emotion regulation, hardiness

## Abstract

Over the last two and a half decades, there has been a surge of research into adverse childhood experiences (ACEs). ACEs have been found to be a significant predictor of mental health outcomes in adulthood, and researchers have begun to explore the relationship between ACEs and mental health outcomes in athletes. However, to the best of the authors’ knowledge, no study has directly compared the mental health of student-athletes to non-student-athletes in the context of exposure to ACEs. In the present study, we compared psychological outcomes observed in college students (123 student-athletes and 149 non-student-athletes) on two mental health variables—depressive symptoms and difficulties in emotion regulation—at one university in the U.S. Results indicated that exposure to ACEs had a significant effect on both depressive symptoms and difficulties in emotion regulation, while student-athlete status only had a significant effect on depressive symptoms. There was no significant interaction effect between exposure to ACEs and student-athlete status. This pattern of main effects and an absence of an interaction effect remained even when the psychological trait of hardiness was controlled for. The implications and limitations of these results will be discussed.

## 1. Introduction

In their seminal study, Felitti and colleagues [1] demonstrated the far-reaching adult health outcomes associated with adverse childhood experiences (ACEs). This initial inquiry sparked a line of research that has produced a plethora of studies in sociology, psychology, and public health investigating ACEs and their correlates [2,3,4]. In the years since Felitti et al.’s [1] investigation into ACEs and adult health outcomes, two findings have consistently been observed in the literature: (1) ACEs are pervasive, and (2) there appears to be a dose-response relationship between ACEs and negative health outcomes in adulthood. Felitti et al. [1] initially found that more than half (52%) of respondents reported at least one ACE in their study. More recently, Merrick et al. [5] found that in a sample of 140,000 individuals across 25 states in the U.S., 60.9% of participants experienced at least one type of ACE and 15.6% reported four or more. Some particularly striking results from Felitti et al.’s [1] study were that individuals who had experienced four or more ACEs were 4 to 12 times more likely to experience alcoholism, drug abuse, depression, and suicide attempts when compared to participants who had experienced no ACEs [1]. Research has continued to demonstrate that with increased ACEs, individuals are at greater risk for substance abuse, depression and anxiety, and suicide [6,7,8]. As such, it appears that ACEs are playing a significant role in the mental health of U.S. adults.

There has been a paucity of literature related to ACEs and student-athlete mental health outcomes. Research exploring the relationship between ACEs and student-athlete mental health outcomes may help inform practice for sport psychology practitioners, coaches, mental health therapists, educators, and other professionals who may work closely with both student-athletes and non-student-athletes. The results of such research may help to create a positive environment for the psychosocial development of individuals across their academic and athletic experiences.

### 1.1. Adverse Childhood Experiences (ACEs)

ACEs traditionally encompass one’s exposure to events such as physical, sexual, or emotional abuse; physical or emotional neglect; and household dysfunction (e.g., witnessing domestic violence). To reiterate and expand on the correlations between health outcomes and ACEs that were mentioned earlier, research has indicated that the more ACEs one is exposed to in childhood, the more likely they are to endorse problematic alcohol use [1,9], drug abuse [1,9], depression [1,9,10], suicide attempts [1,9], smoking, poor self-rated health, more than 50 sexual intercourse partners, sexually transmitted diseases, physical inactivity, severe obesity, ischemic heart disease, cancer, chronic lung disease, skeletal fractures, liver disease [1], chronic fatigue, and sleep problems [10]. This wide array of adult health concerns that have been correlated with ACEs has led to some scholars stating that “at least a third of mental and behavioral disorders can be attributed to ACEs” [4] (p. 182).

Danese and McEwen [11] suggest that stressful childhood experiences may impact biological processes, affecting physiological responses to stress in adulthood. The human body has evolved to maintain stability through adaptation of the nervous, endocrine, and immune systems in response to environmental and physiological changes. The process by which the body seeks to preserve homeostasis through changes in its functioning is known as allostasis [11]. Psychosocial stressors (e.g., a psychologically abusive parent) may induce changes in these highly integrated allostatic systems (e.g., initiation of the “fight-or-flight” response), which in the short term may be adaptive and functional to manage a stressor but detrimental to physical and mental health in the long term if allostatic systems are chronically activated. Indeed, such repeated and sustained exposure to psychosocial stressors has been linked to detrimental physiological outcomes [11]. This “wear and tear” due to chronic stress is known as allostatic load [11].

In childhood, ACEs may cause prolonged physiological responses in allostatic systems, increasing allostatic load and negatively impacting physiological and psychological development. However, Danese and McEwen [11] point out that many factors may affect the outcomes observed in adulthood following ACE exposure. Namely, (1) the nature of the childhood adversity (e.g., when the ACE(s) occurred, what type of ACE(s) were experienced, the number of times an ACE or ACEs was/were endured, etc.), (2) individual resilience and vulnerability factors such as one’s genetics and previous experiences, and (3) even though ACEs can affect the development of multiple allostatic systems (multifinality), any number of factors could have similar effects (equifinality), which may decrease or increase the relationship between ACEs and allostatic load [11].

Involvement in athletics may represent one such factor that could serve to buffer or potentiate the relationship between ACEs and allostatic load. Athletics may protect against ACEs due to the opportunities to engage in regular exercise, get involved with a group of peers (possibly increasing social support), have adult mentors (e.g., coaches), and/or engage with a pleasurable activity. However, these same factors could potentiate the relationship between ACEs and allostatic load should individuals experience a negative team culture (e.g., bullying), have hypercritical coaches, feel undue pressure to perform at a high level, and/or experience stress due to the time demands placed on them as they advance through sport. Researching how involvement with sport into adulthood is related to mental health, particularly if individuals have been exposed to ACEs, may help illuminate if such a buffering or potentiating relationship exists.

### 1.2. Depressive Symptoms and Difficulties in Emotion Regulation

When researching collegiate student-athletes and non-student-athletes, two psychological outcomes that are relevant to both ACEs and the student-athlete literature are depression/depressive symptoms and difficulties in emotion regulation. Depression is the second most common mental health concern college students report ever being diagnosed with [12] and the positive correlation between ACEs and depressive symptoms in adulthood has been well-established in the literature [1,13]. In fact, in 2020, the Centers for Disease Control in the United States suggested over 2 million cases of depression might be prevented through targeting ACEs [4]. Research comparing collegiate student-athletes to non-student-athletes [14,15] has found student-athletes to display lower levels of depression than non-student-athletes. However, this research has not taken into consideration the ACEs participants may have experienced, which could further contextualize results.

ACEs have also been found to positively predict difficulties in emotion regulation [16]. This relationship is noteworthy because multiple studies have suggested that emotion regulation ability plays a significant, mediating role between the ACEs one experiences in childhood and the psychological distress they report in adulthood [16,17,18]. Interestingly, when comparing collegiate student-athletes to non-student-athletes, student-athletes appear to have less difficulty regulating their emotions [15,19]. Athletics has the potential for helping youth to develop psychological well-being through a variety of means, such as requiring regular physical exercise, increasing social connections/support, and providing ample opportunity to practice regulating emotions in the face of challenging situations. What remains unclear, though, is if ACEs impact the mental health of student-athletes differently than non-student-athletes and/or if athletic involvement may provide a buffer to childhood adversities not observed in non-student-athlete populations. Therefore, athletic involvement appears to decrease the likelihood of experiencing depressive symptoms and increase the ability to regulate emotions in collegiate student-athletes, but it is not clear if this relationship is maintained when examining student-athletes and non-student-athletes who have a history of ACEs.

### 1.3. ACEs and Student-Athletes

Given the pervasiveness of ACEs, it would seem likely a significant proportion of student-athletes have experienced one or more ACEs. Indeed, in a sample of 477 student-athletes from 55 different colleges/universities, it was found that 64.5% of the sample had experienced at least one ACE and 38.7% experienced two or more [20]. However, in a separate study of 304 collegiate student-athletes, Kaier et al. [21] found only 14% of their sample reported a single ACE and only 17% reported multiple ACEs. Just as with non-athletes, research appears to demonstrate negative psychological health outcomes for student-athletes who have experienced ACEs, particularly athletes who have experienced multiple ACEs. The literature examining the consequences of ACEs in student-athletes has found ACEs are associated with increased risk for anxiety, depression, perceived stress, somatization disorder, substance use, insomnia, and prescription medication use, as well as decreased social support, sleep quality, and sleep [20,21,22]. Despite these outcomes, research with high-level student-athletes also seems to indicate that there is a protective factor associated with sport involvement. Kaier et al. [21] found there to be a buffering effect associated with sport involvement for up to one ACE for collegiate student-athletes. The authors [21] speculated that regular exercise (which is a requisite of high-level athletics) may provide a protective factor to allostatic load, leading to collegiate student-athletes having a buffer to ACEs while developing as youth. Kaier et al. [21] recommended exploration into potential mechanisms that may contribute to the relationship between ACEs, allostatic load, and health outcomes. Additionally, they [21] recommended that direct comparisons with high-level athletes and non-athletes (e.g., collegiate student-athletes and non-student-athletes) in the context of ACEs research could be beneficial to ascertain if high-level athletics is a resilience factor. In accordance with these suggestions, one construct worth investigating in ACEs, student-athletes, and mental health research is hardiness.

### 1.4. Hardiness

Hardiness is a personality trait that may help researchers and practitioners better understand individuals’ health outcomes in the wake of stressful life events. Kobasa [23] found that individuals high on hardiness were able to experience high amounts of life stress and were less likely to experience illness than individuals who were low on hardiness. This finding has been replicated for general mental health as well—the lower one’s hardy attitudes are, the greater the likelihood of psychopathology is (e.g., depression, anxiety, post-traumatic stress disorder [24,25]. Kobasa et al. [26] outlined the “3C’s” of hardiness as follows:

(1) Commitment: Someone’s tendency to involve themselves in whatever they are doing and encounter (rather than alienate themselves).
(2) Control: The tendency to feel and act as if one is influential rather than helpless when confronting the various contingencies of life.
(3) Challenge: Individuals believe that change rather than stability is normal in life, and anticipating changes is an incentive for growth instead of a threat to security.

Hardiness has been associated with resilience, stress-related growth, good health, and performance under stressful conditions [23,24,27,28,29,30]. Of these associations, a protective factor that has been of particular interest in the mental health literature is resilience. Resilience is one’s ability to experience a highly stressful life event but still be able to maintain healthy psychological and physical functioning after the event. Thus, resilience is a process by which individuals attempt to effectively cope with stressful situations [31,32]. Hardiness, however, is a set of personality characteristics that help guide one’s decision-making when handling stressful life events and is generally considered a dispositional response style to stress. Therefore, possessing hardy characteristics may help someone be more likely to act resiliently when faced with stressful life events [24,29]. One notion of the student-athlete experience is that participation in athletics may promote resilience in individuals through having to cope with adversity that is inherent in sport. It is worth investigating if endorsement of hardy attitudes (i.e., a pathway to resilience; [24,29]) is indeed promoted through sport participation up into adulthood and thereby may increase student-athletes’ resilience to adverse situations (e.g., ACEs).

To date, direct comparisons of hardy attitudes between athletes and non-athletes are minimal, and results are mixed. Skirka [33] found athletes to score higher on hardiness than non-athletes. However, Kilchrist [34] found individual athletes to be significantly lower in the challenge dimension of hardiness when compared to non-athletes and team-sport athletes and found no significant differences in total hardiness score between contact athletes, non-contact athletes, and non-athletes. Given the limited research in this area, scholarship that takes into consideration the endorsement of hardy attitudes by student-athletes and non-student-athletes, as well as the ACEs they have experienced, may provide a more comprehensive understanding of mental health outcomes observed within each group when making comparisons between them.

### 1.5. Present Study

As mentioned, Kaier et al. [21] suggested that it would be valuable for researchers to directly compare ACEs and adult health outcome data between college student-athletes and non-student-athletes in order to draw inferences about potential resilience factors. Given this recommendation and the authors’ interest in mental health, we chose to examine the associations between ACEs and student-athlete status with depressive symptoms and difficulties in emotion regulation, two mental health variables shown to be positively associated with ACEs [17,20].

The current study had four objectives. The first objective was to compare depressive symptoms and difficulties in emotion regulation in college students with varying levels of ACEs exposure. The second objective was to compare depressive symptoms and difficulties in emotion regulation in college student-athletes and non-student-athletes. Third, we wanted to test if ACEs and student-athlete status would uniquely interact when examining depressive symptoms and difficulties in emotion regulation in adulthood. Finally, should relationships exist between ACEs and student-athletes’ status with depressive symptoms and difficulties in emotion regulation, our fourth objective would be to examine how controlling for hardiness might impact those relationships. Given these objectives, we had four hypotheses:

**Hypothesis** **1:** *There will be a significant main effect on participants’ depressive symptoms and difficulties in emotion regulation based on the number of ACEs they report*.

**Hypothesis** **2:** *There will be a significant main effect on participants’ depressive symptoms and difficulties in emotion regulation based on their student-athlete status*.

**Hypothesis** **3:** *There will be a significant interaction between the number of ACEs one experienced and their student-athlete status on depressive symptoms and difficulties in emotion regulation*.

**Hypothesis** **4:** *If significant main effects or an interaction effect are found, controlling for hardiness will lead to there no longer being a significant main effect and/or interaction effect*.

## 2. Materials and Methods

### 2.1. Participants

The study was conducted according to the Declaration of Helsinki [35] and was approved by the university Institutional Review Board (IRB). A total of 243 participants were recruited for the current study from a university in the U.S. Midwest that competed at the NCAA Division II level. Informed consent was obtained from all participants in the study. Data was collected from two sources. The majority of student-athlete data was collected through convenience sampling via surveys that were emailed to student-athletes. Student-athletes were paid USD 5.00 for their participation in the study by way of grant funding provided by the Association of Applied Sport Psychology. During the same semester, a separate study exploring the impact of ACEs on college students was being conducted through an Introduction to Psychology research pool. This archived data was later used for comparison with student-athlete data collected for the present study. Data was examined to ensure that there were no duplicate participants and for the purposes of separating student-athlete data from non-student-athlete data. In total, there were 272 participants. There were 123 student-athletes (45.22%) who participated in the study. Student-athletes were determined in the survey email by asking participants, “Are you currently a college student-athlete?” If they responded “yes,” we then asked them what sport/team they participated in/on. Student-athlete data was obtained for the Introductory Psychology research pool participants by using institutional data. We then cross-checked the teams that students were reported to be on with the university athletic department’s list of athletic teams. This resulted in the inclusion of student-athletes from 19 sports/teams, which included men’s baseball, women’s softball, men’s and women’s basketball, men’s and women’s soccer, men’s and women’s track and field, men’s and women’s cross country, cheer team, dance team, men’s and women’s golf, men’s and women’s tennis, men‘s and women‘s soccer, and women’s volleyball. Of the 123 student-athletes, 111 (90.24%) came from the survey emails and 12 (9.76%) came from the Introduction to Psychology research pool. There was data for 149 non-student-athletes (54.78%) from the Introduction to Psychology research pool. Out of the total sample, participants’ ages ranged from 18 to 29 years old (*M* = 19.51, *SD* = 1.67), and 169 participants identified as female (62.13%), while 103 identified as male (37.87%). The sample was predominantly White or Caucasian (*n* = 244, 89.71%), with 10 individuals identifying as Black (3.68%), 6 identifying as Hispanic/Latin(x) (2.21%), 6 identifying as Asian or Asian American (2.21%), 2 identifying as American Indian or Alaskan Native (0.74%), 2 identifying as Multiracial (0.74%), 1 identifying as Hawaiian or Other Pacific Islander (0.37%), and data on racial/ethnic identity was not specified for one individual (0.37%). Finally, students reported being freshmen (*n* = 140, 51.47%), sophomores (*n* = 67, 24.63%), juniors (*n* = 44, 16.18%), seniors (*n* = 17, 6.25%), graduate students (*n* = 3, 1.10%), and academic class data was unspecified for 1 student (0.37%).

### 2.2. Measures

#### 2.2.1. Adverse Childhood Experiences (ACE) Study Questionnaire

The ACE Study questionnaire was developed by Felitti et al. [1] to examine how the relationship between negative childhood experiences may be related to health risk factors later in life. The ACE questionnaire consists of 10 categories that are comprised of 17 questions. Those 10 categories are emotional abuse (2 questions), physical abuse (2 questions), sexual abuse (2 questions), emotional neglect (2 questions), physical neglect (2 questions), exposure to household substance abuse (1 question), exposure to household mental illness (1 question), exposure to violent treatment of mother or stepmother (3 questions), parental separation or divorce (1 question), and exposure to criminal behavior (1 question). Sample items include “Did a household member go to prison?” and “Was a household member depressed or mentally ill, or did a household member attempt suicide?” Participants respond to questions with either “yes” or “no” responses (yes = 1, no = 0). In order to score the measure, the sum of exposures to the 10 categories is found. Therefore, the range of the measure is 0 (no exposure to ACEs) to 10 (exposure to all categories of ACEs; [36]). The ACE Study questionnaire has demonstrated acceptable reliability in past research [21]. Similarly, in the present study, internal consistency was high (*a* = 0.77).

#### 2.2.2. Dispositional Resilience Scale

The Dispositional Resilience Scale is a 15-item measure (DRS-15) of hardiness, measuring one’s likelihood to respond in a resilient manner to stressors. The DRS-15 consists of 3 subscales (Commitment, Control, and Challenge), with 5 items each [28]. Participants respond to statements such as, “By working hard you can nearly always achieve your goals,” and, “My choices make a real difference in how things turn out in the end,” on a 4-point Liker scale, indicating how true a statement is for them from 0 (not at all true) to 3 (completely true). Participant scores are summed after negatively keyed items are reverse-scored. Thus, subscale scores range from 0 to 15, with higher scores indicating greater endorsement of a particular hardiness characteristic (i.e., commitment, control, or challenge), and total scores range from 0 to 45, with higher scores indicating greater composite hardiness [28].

The DRS-15 has demonstrated criterion-related validity across several samples [27,37] as well as high test-retest reliability over 3 weeks (*r* = 0.78; [28]). The DRS-15 has been found to be positively correlated with measures of resilience in two samples (*r* = 0.60 and *r* = 0.67; [38]) as well as negatively correlated with measures of depression (*r* = −0.44, *r* = −0.40), state anxiety (*r* = −0.44, *r* = −0.29), and trait anxiety (*r* = −0.52, *r* = −0.47) in those samples [38]. In the present study, each subscale demonstrated acceptable internal consistency—commitment (*a* = 0.81), control (*a* = 0.67), and challenge (*a* = 0.69)—as did the total scale when all items were considered collectively (*a* = 0.76).

#### 2.2.3. Patient Health Questionnaire

The Patient Health Questionnaire is a 9-item measure (PHQ-9) that is widely used by health professionals to assess depression severity through endorsement of depressive symptoms by questionnaire respondents [39]. The PHQ-9 consists of statements regarding symptoms of depression (e.g., “Little interest or pleasure in doing things,” “Feeling down, depressed, or hopeless”), to which respondents indicate how often they have experienced the specified symptoms over the past two weeks on a 4-point scale ranging from 0 (not at all) to 3 (nearly every day), resulting in a total score range of 0–27, with higher scores indicating greater endorsement of depressive symptoms. Responses to items are summed, and total scores of 5, 10, 15, and 20 serve as cut points for mild, moderate, moderately severe, and severe depression, respectively [39]. The test-retest reliability of the PHQ-9 over a 48-h period is high (*r* = 0.84; [39]). The PHQ-9 has been shown to correlate positively with other measures of depression, such as the Center for Epidemiologic Studies of Depression 10 Scale (*r* = 0.80; [40]), as well as correlate negatively with psychological well-being (*r* = −0.65; [40]). In the present study, the PHQ-9 had a high level of internal consistency (*α* = 0.91).

#### 2.2.4. Difficulties in Emotion Regulation Scale

The Difficulties in Emotion Regulation Scale (DERS) is a 36-item self-report measure that assesses clinically relevant difficulties in emotion regulation [41,42]. The DERS is comprised of six dimensions: (1) Lack of Emotional Awareness (6 items), (2) Lack of Emotional Clarity (5 items), (3) Difficulties Controlling Impulsive Behaviors When Distressed (6 items), (4) Difficulties Engaging in Goal-Directed Behavior When Distressed (5 items), (5) Nonacceptance of Negative Emotional Responses (6 items), and (6) Limited Access to Effective ER Strategies (8 items). Sample items include, “When I am upset, I have difficulty focusing on other things,” and, “I am attentive to my feelings.” Participants respond to items on a 5-point Likert scale ranging from 1 (almost never) to 5 (almost always). Items are summed to provide both subscale scores and total DERS scores, with higher scores indicating greater difficulties in regulating emotions.

The DERS has demonstrated high test-retest reliability for a 4–8 week period (*ρ*_I_ = 0.88; [41]). Research conducted with adults with severe psychopathology has found total scores on the DERS to correlate positively with experiential avoidance (*r* = 0.70), depressive symptoms (*r* = 0.45), anxiety symptoms (*r* = 0.44), and somatic complaints (*r* = 0.28; [43]). The DERS has been found to be negatively correlated with psychological well-being (*r* = −0.38; [44]). In the present study, the DERS demonstrated high internal consistency for the total scale (*α* = 0.94), as well as each of the subscales: (1) Lack of Emotional Awareness (*α* = 0.86), (2) Lack of Emotional Clarity (*α* = 0.81), (3) Difficulties Controlling Impulsive Behaviors When Distressed (*α* = 0.85), (4) Difficulties Engaging in Goal-Directed Behavior When Distressed (*α* = 0.88), (5) Nonacceptance of Negative Emotional Responses (*α* = 0.91), and (6) Limited Access to Effective Emotion Regulation Strategies (*α* = 0.90).

### 2.3. Data Analysis

We first examined descriptive statistics for the study’s main variables. Additionally, we conducted a chi-square analysis to assess if there was a significant association between student-athlete status and the number of ACEs participants reported. However, the primary data analyses for the present study were multiple analysis of variance (MANOVA) and multiple analysis of covariance (MANCOVA). In the MANOVA, student-athlete status and the number of ACEs a participant endorsed (i.e., zero, one, or multiple) were entered as independent variables, and difficulties in emotion regulation and depressive symptoms were entered as dependent variables. In the MANCOVA, the independent and dependent variables remained the same, however, hardiness was added as a covariate. Follow-up univariate analyses of variance (ANOVA) and analyses of covariance (ANCOVA) would be conducted for any main effects or interactions that may be observed in the MANOVA and MANCOVA models.

Multivariate and univariate assumptions for the purposes of the MANOVA and MANCOVA were tested. Assumptions of the MANOVA model were checked prior to analyses. There was a linear relationship between the dependent variables within each group of the independent variables as assessed by a scatterplot. There was no evidence of multicollinearity as assessed by Pearson correlation (|*r*| < 0.9). There were three outliers for depressive symptoms (two student-athletes and one non-student-athlete) and one outlier for difficulties in emotion regulation (one student-athlete), as assessed by inspection of boxplots. However, there were no multivariate outliers in the model as assessed by Mahalanobis distance (*p* > 0.001). Given that there were no multivariate outliers and that the data were plausible data points that did not represent entry errors or truly unusual data points, we chose to leave the univariate outliers in the dataset. When testing for multivariate normality, there were some slight deviations in normality as assessed by Kolmogorov–Smirnov or Shapiro–Wilk tests, depending on group size (i.e., Kolmogorov–Smirnov was used for groups with more than 50 participants, and Shapiro–Wilk was used for groups with less than 50 participants). Specifically, when using the Kolmogorov–Smirnov test, violations in normality occurred when assessing depressive symptoms in the student-athlete, no ACEs group (*p* < 0.001), and the non-student-athlete, no ACEs group (*p* < 0.001). Additionally, using Shapiro–Wilk’s test, violations in normality were found when measuring depressive symptoms in the student-athlete, one ACE group (*p* < 0.001), and the non-student-athlete, one ACE group (*p* = 0.03). Despite these deviations in normality, we chose to carry on with the analysis without transforming our data due to the two-way MANOVA being robust to violations of normality [45]. There was homogeneity of covariance matrices, as assessed by Box’s M test (*p* = 0.066). Within the model, there was homogeneity of variance for difficulties in emotion regulation (*p* = 0.357), but not for depressive symptoms (*p* = 0.042), as assessed by Levene’s test of homogeneity of variances. However, given that there was homogeneity of covariance matrices for this multivariate model and that the univariate analyses that would be conducted are robust to violations of normality (e.g., ANOVA; [46]), which may impact homogeneity of variance, we chose to continue with analyses without transforming data.

Follow-up tests using MANCOVA were planned, and assumptions specific to MANCOVA were checked once the covariate, hardiness, was added to the model. There was a linear relationship between the covariate, hardiness, and each dependent variable within each group of the independent variable, which was again assessed by inspection of scatterplots. There was no evidence of multicollinearity as assessed by Pearson correlation (|*r*| < 0.9). There was homogeneity of regression slopes as assessed by the interaction terms between the dependent variables and the covariate *F*(10, 492) = 0.81, *p* = 0.621. There was homoscedasticity within each combination of groups of the two independent variables, as assessed by inspection of the studentized residuals plotted against the predicted values for each dependent variable within each group. There was one univariate outlier on depressive symptoms as assessed by a standardized residual greater than 3 standard deviations. However, this appeared to be a valid data point, thus it was retained in the data set. There were no multivariate outliers as assessed by Mahalanobis distance (*p* > 0.001). When evaluating for multivariate normality through examination of residuals, there was one slight deviation in normality for depressive symptoms as assessed by Kolmogorov–Smirnov’s test (*p* = 0.003). There was homogeneity of covariances as assessed by Box’s M test (*p* = 0.075). Similar to the MANOVA model, Levene’s test for equality of error variances was used to assess homogeneity of variance for univariate analyses. Again, the assumption of homogeneity of variance was met for difficulties in emotion regulation (*p* = 0.205) but not depressive symptoms (*p* = 0.003). Once more, due to the robust univariate analyses that would be used (e.g., ANCOVA), it was decided to not transform the data and continue with the analyses. For both the MANOVA and MANCOVA, partial *η*^2^ was used as the measure of effect size. Using Cohen’s [47] guidelines, values of 0.01 were considered small, 0.06 were considered medium, and 0.14 were considered large. All post hoc comparison tests were carried out using Bonferroni correction. Alpha was set at 0.05 for significance testing.

## 3. Results

**Figure 1:**
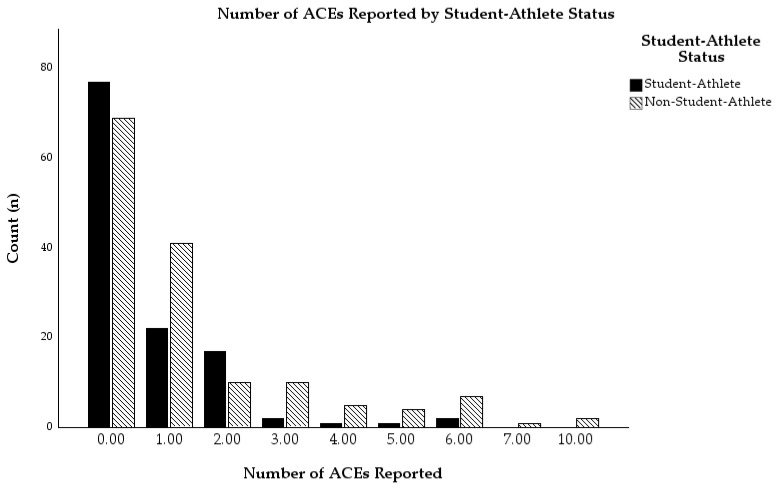
Number of ACEs reported by student-athlete status.

**Figure 2:**
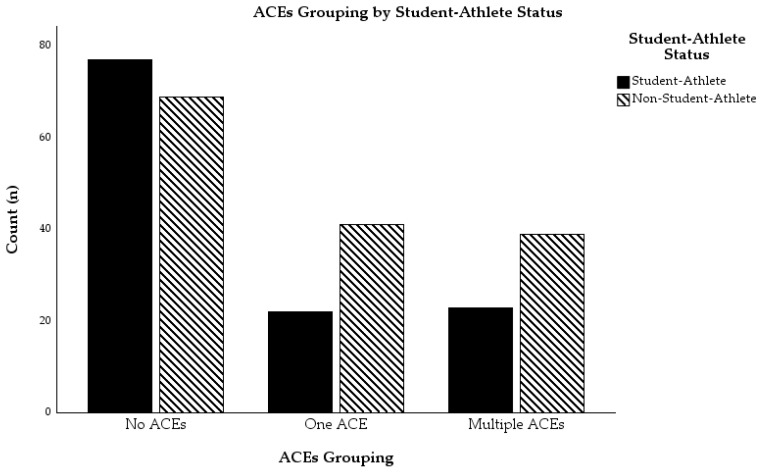
ACEs grouping of participants by student-athlete status.

Descriptive statistics pertaining to depressive symptoms, difficulties in emotion regulation, and hardiness can be found in Table 1. Rather than use the traditional ACEs grouping categories (i.e., 0, 1, 2, 3, and 4+ ACEs; [1]), we chose to follow the approach taken by Kaier et al. [21] and divided participants up into three ACEs groupings: no ACEs (*n* = 146), one ACE (*n* = 63), and multiple ACEs (*n* = 62). This strategy also increased subgroup sizes so that we were better able to make comparisons between subgroups of student-athletes and non-student-athletes than if we used traditional grouping methods. Using this approach, there were 77 student-athletes and 66 non-student-athletes in the no ACEs group, 22 student-athletes and 41 non-student-athletes in the one ACE group, and 23 student-athletes and 39 non-student-athletes in the multiple ACEs group. There was a statistically significant association between student-athlete status and ACEs grouping, *x*^2^(2) = 7.68, *p* = 0.021; however it was a relatively weak association, Cramer’s *V* = 0.17 [47]. Results from the chi-square appear to indicate that student-athletes were less likely to experience ACEs than non-student-athletes. For a visual representation of the number of ACEs reported broken down by student-athlete status, see Figure 1. To see participants’ ACEs grouping broken down by student-athlete status, see Figure 2.

**Figure 3:**
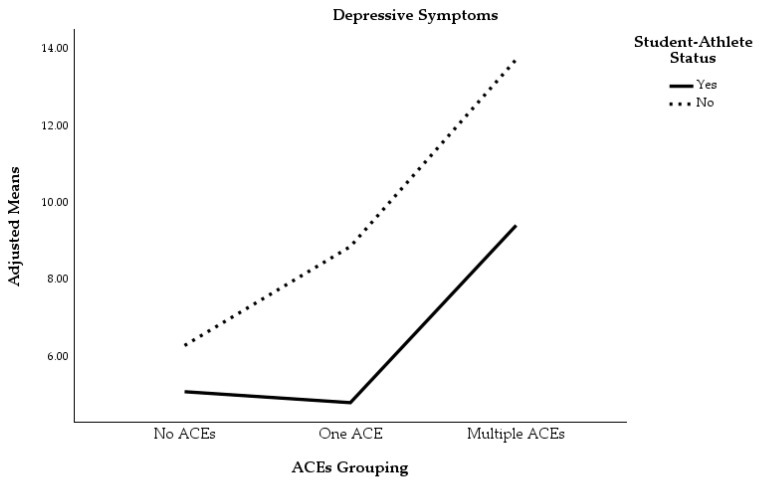
Depressive symptoms by ACEs grouping and student-athlete status.

**Figure 4:**
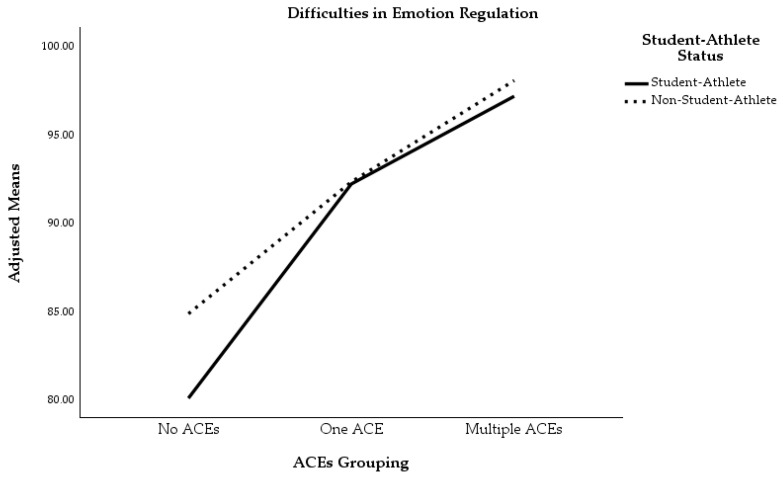
Difficulties in emotion regulation by ACEs grouping and student-athlete status.

We first conducted a two-way MANOVA with ACEs grouping and student-athlete status entered as the independent variables and difficulties in emotion regulation (DERS) and depressive symptoms (PHQ-9) entered as the dependent variables. There was a total of 260 participants included in this analysis. There was a statistically significant main effect between participants on the combined dependent variables based on ACEs grouping, *F*(4, 506) = 11.47, *p* < 0.001, Wilks’ Λ = 0.84, partial *η*^2^ = 0.08, 1 − *β* = 1.00, as well as student-athlete status, *F*(2, 253) = 9.85, *p* < 0.001, Wilks’ Λ = 0.93, partial *η*^2^ = 0.07, 1 − *β* = 0.98. Additionally, there was a statistically significant interaction effect between ACEs grouping and student-athlete status on the combined dependent variables, *F*(4, 506) = 2.44, *p* = 0.046, Wilks’ Λ = 0.96, partial *η*^2^ = 0.02, 1 − *β* = 0.70. Interestingly, though, when univariate interaction effects were examined, no significant interactions between ACEs grouping and student-athlete status were observed for depressive symptoms, *F*(2, 254) = 2.01, *p* = 0.137, partial *η*^2^ = 0.02, or difficulties in emotion regulation, *F*(2, 254) = 0.32, *p* = 0.730, partial *η*^2^ = 0.00. This lack of an interaction effect indicates that the adverse outcomes associated with increased ACEs exposure occurred at relatively the same rate for student-athletes and non-student-athletes (see Figure 3 and Figure 4 for graphical representations).

Follow-up univariate ANOVAs showed that depressive symptoms *F*(2, 254) = 19.89, *p* < 0.001, partial *η*^2^ = 0.14, and difficulties in emotion regulation *F*(2, 254) = 11.27, *p* < 0.001, partial *η*^2^ = 0.08 were significantly different between participants of different ACEs groupings. Individuals in the multiple ACEs group reported significantly more depressive symptoms (*M* = 11.59, *SE* = 0.79) than individuals in the one ACE (*M* = 6.85, *SE* = 0.77, *p* < 0.001) and no ACEs (*M* = 5.70, *SE* = 0.50, *p* < 0.001) groups. There was no statistically significant difference between the one ACE and no ACEs groups (*p* = 0.646) as it pertained to endorsement of depressive symptoms. With regard to difficulties in emotion regulation, individuals in the multiple ACEs group (*M* = 97.68, *SE* = 2.89) reported significantly greater difficulty in regulating emotions than individuals in the no ACEs group (*M* = 82.54, *SE* = 1.82, *p* < 0.001). Participants in the one ACE group (*M* = 92.33, *SE* = 2.82) reported significantly greater difficulty in emotion regulation than participants in the no ACEs group (*p* = 0.012). There was no statistically significant difference between participants in the multiple ACEs group and the one ACE group with regard to difficulties in emotion regulation (*p* = 0.559).

Follow-up univariate ANOVAs demonstrated that there was a statistically significant main effect for student-athlete status on endorsement of depressive symptoms, *F*(1, 254) = 15.53, *p* < 0.001, partial *η*^2^ = 0.06, but not for difficulties in emotion regulation, *F*(1, 254) = 0.43, *p* = 0.514, partial *η*^2^ = 0.00. Student-athletes reported fewer depressive symptoms (*M* = 6.45, *SE* = 0.64) than non-student-athletes (*M* = 9.64, *SE* = 0.50, *p* < 0.001).

A two-way MANCOVA was conducted to follow-up the MANOVA results. There was a total of 259 participants included in this analysis. Results of the two-way MANCOVA demonstrated that there were statistically significant main effects for ACEs groupings on the combined dependent variables after controlling for hardiness, *F*(4, 502) = 8.98, *p* < 0.001, Wilks’ Λ = 0.87, partial *η*^2^ = 0.07, 1 − *β* = 0.99, as well as for student-athlete status, *F*(2, 251) = 7.17, *p* < 0.001, Wilks’ Λ = 0.95, partial *η*^2^ = 0.05, 1 − *β* = 0.93. The covariate, hardiness, also had a significant association with the combined dependent variables *F*(2, 251) = 34.57, *p* < 0.001, Wilks’ Λ = 0.78, partial *η*^2^ = 0.22, 1 − *β* = 1.00. Finally, there was no statistically significant interaction for ACEs grouping and student-athlete status on the combined dependent variables after controlling for hardiness, *F*(4, 502) = 2.21, *p* = 0.067, Wilks’ Λ = 0.97, partial *η*^2^ = 0.02, 1 − *β* = 0.65. Collectively, these results indicate that controlling for hardiness did not significantly alter the relationships that were observed between ACEs grouping and student-athlete status with the combined dependent variables in the MANOVA model.

Follow up univariate ANCOVAs were conducted for ACEs grouping. There were statistically significant differences for both depressive symptoms, *F*(2, 252) = 15.98, *p* < 0.001, partial *η*^2^ = 0.11, and difficulties in emotion regulation, *F*(2, 252) = 5.78, *p* = 0.004, partial *η*^2^ = 0.04, based on ACEs grouping. Depressive symptoms were significantly greater in the multiple ACEs group (*M* = 11.12, *SE* = 0.74) than they were in either the one ACE (*M* = 6.52, *SE* = 0.71, *p* < 0.001) or no ACEs (*M* = 6.30, *SE* = 0.47, *p* < 0.001) groups. There was no significant difference between the one ACE and no ACEs groups (*p* = 1.000) as it pertained to depressive symptoms. Difficulties in emotion regulation were greater in the multiple ACEs group (*M* = 95.20, *SE* = 2.69) than in the no ACEs group (*M* = 84.83, *SE* = 1.69, *p* = 0.004). There was no statistically significant difference in emotion regulation between the multiple ACEs group and the one ACE group (*M* = 91.05, *SE* = 2.57, *p* = 0.793) or the one ACE group and the no ACEs group (*p* = 0.136). The lack of a significant difference between the one ACE group and the no ACE group with regard to difficulties in emotion regulation represents a difference between the MANOVA and MANCOVA models, as there was a statistically significant difference between these two groups within the MANOVA.

Follow-up univariate ANCOVAs demonstrated that there was a statistically significant main effect for student-athlete status on endorsement of depressive symptoms, *F*(1, 252) = 9.53, *p* = 0.002, partial *η*^2^ = 0.04, but not for difficulties in emotion regulation, *F*(1, 252) = 0.12, *p* = 0.727, partial *η*^2^ = 0.00. Student-athletes reported fewer depressive symptoms (*M* = 6.82, *SE* = 0.59) than non-student-athletes (*M* = 9.14, *SE* = 0.46, *p* = 0.002). The results of the MANOVA, MANCOVA, and follow-up ANOVAs and ANCOVAs have been summarized in Table 2 and Table 3.

Finally, an ancillary analysis was conducted following the MANCOVA to investigate potential group differences in hardiness via a two-way ANOVA. Within the ANOVA, ACEs grouping and student-athlete status were entered as the independent variables, and hardiness was entered as the dependent variable. Prior to interpreting the results of the ANOVA, assumptions for univariate analysis were checked given the new dependent variable of hardiness. The data was found to be normally distributed, free of outliers, and to have homogeneity of variances, thus meeting the assumptions for univariate analysis. There were 264 participants included in this analysis. Results of the ANOVA indicated a significant main effect for both ACEs grouping, *F*(2, 258) = 6.44, *p* = 0.002, partial *η*^2^ = 0.05, and student-athlete status, *F*(1, 258) = 5.47, *p* = 0.020, partial *η*^2^ = 0.02. There was not a statistically significant interaction between ACEs grouping and student-athlete status, *F*(2, 258) = 1.70, *p* = 0.184, partial *η*^2^ = 0.01. Participants in the multiple ACEs grouping scored significantly lower on hardiness (*M* = 27.50, *SE* = 0.73) than individuals in the no ACEs group (*M* = 30.23, *SE* = 0.45, *p* = 0.005). Individuals in the one ACE group (*M* = 28.14, *SE* = 0.71) also scored significantly lower on hardiness than individuals in the no ACEs group (*p* = 0.042). There were no significant differences between the multiple ACEs and one ACE groups (*p* = 1.000). Student-athletes scored significantly higher on hardiness (*M* = 29.49, *SE* = 0.59) than non-student-athletes (*M* = 27.76, *SE* = 0.46, *p* = 0.020).

## 4. Discussion

The primary purpose of the current study was to compare depressive symptoms and difficulties in emotion regulation in college student-athletes versus non-student-athletes, while accounting for the number of ACEs participants experienced. Our first hypothesis was correct, and there was a significant main effect for the number of ACEs participants endorsed. Individuals who endorsed multiple ACEs reported significantly greater depressive symptoms than individuals who endorsed only one ACE or no ACEs. Participants in the multiple ACEs and the one ACE group also reported significantly greater difficulty in emotion regulation than participants in the no ACEs grouping. These results align with the general trend that has been observed for both athletes and non-athletes, which is, the more ACEs one is exposed to in childhood, the more likely they are to experience negative mental health outcomes in adulthood [1,13,21]. Our second hypothesis was partially supported by the data. Student-athletes reported significantly fewer depressive symptoms than non-student-athletes, but there was not a significant difference between these two groups when examining differences in difficulties in emotion regulation. Again, this result aligns with prior research indicating that student-athletes generally demonstrate lower levels of depression than non-student-athletes [14], possibly due to the myriad of ways in which athletics may promote mental health during development (e.g., increased social supports, regular exercise, etc.).

Our third hypothesis was not supported by the data. Within the MANOVA, there was a significant interaction effect between the number of ACEs participants endorsed and student-athlete status for the combined dependent variables; however, when follow-up univariate ANOVAs were examined, there were no statistically significant interaction effects between ACEs grouping and student-athlete status on either dependent variable. Increased exposure to ACEs did not have a differential effect on mental health when examining non-student-athlete mental health compared to student-athlete mental health. Therefore, the increased depressive symptoms and difficulties in emotion regulation that were associated with increased ACEs occurred at relatively the same rate, regardless of student-athlete status. As alluded to earlier, athletic involvement may decrease the allostatic load associated with ACEs and promote mental health due to the opportunity for regular exercise [21] and facilitation of other positive psychosocial developments (e.g., increased social support, positive mentors through coaching, promotion of hardy attitudes, etc.), but this may come at a cost when competing at higher levels of sport. In particular, college student-athletes may face stressors such as overtraining [48], burnout [49], high demands on time [50], help-seeking stigma [51], injuries [30], and social media pressure and criticism [52] that their non-student-athlete peers do not. These stressors may counteract the positive benefits of athletic involvement on mental health and, thus, not significantly alter the relationship between ACEs and psychological outcomes.

Our fourth and final hypothesis was also not supported by the data. When hardiness was controlled for, there were still main effects observed for ACEs grouping and student-athlete status on the combined dependent variables, and there was not a statistically significant interaction effect. Differences in depressive symptoms based on ACEs grouping and student-athlete status remained the same as they did in the MANOVA model. However, some slight group differences were observed in the MANCOVA model when comparing differences in difficulties in emotion regulation. There was no longer a significant difference in difficulties in emotion regulation between participants in the one ACE and no ACEs groups once hardiness was controlled for. Ancillary analyses indicated that individuals who reported no ACEs scored higher on hardiness than individuals with one or multiple ACEs, and student-athletes scored higher on hardiness than non-student-athletes. Collectively, despite small group differences, the overall relationships between ACEs and student-athlete status with depressive symptoms and difficulties in emotion regulation were not significantly altered by controlling for hardiness. Therefore, while student-athletes, on average, endorsed higher levels of hardy attitudes, the trait of hardiness alone was not enough to account for the differences in mental health observed between student-athletes and non-student-athletes. Student-athletes may have reported fewer depressive symptoms and difficulties in emotion regulation for a number of reasons beyond simply possessing hardy attitudes, which will be considered in the following section.

### 4.1. Implications

Results of the current study underscore the importance of sport involvement for those working with youth (e.g., teachers, coaches, etc.). Student-athletes appeared less likely to experience ACEs growing up, which may have contributed to them being less likely to experience depressive symptoms than non-student-athletes. Further, sport involvement may have provided exercise regimens [21] and consistent social support [53] that could have decreased allostatic load even when exposed to ACEs, possibly contributing to a buffering effect. Time spent in sporting activities in childhood (e.g., practices, games, etc.) that are necessary to achieve a high level of sport involvement (i.e., collegiate sport) later in life may be likely to lead to time spent away from a negative home environment or situations, possibly decreasing the chance of experiencing ACEs and/or the time exposed to the negative repercussions of ACEs (e.g., family conflict; [21]). This time spent away from potentially negative environments to engage in resilience-promoting activities (e.g., sport, exercise, peer connections) may be beneficial to children’s mental health as they age into adulthood, even if they do experience some ACEs. Thus, it appears that athletic involvement by and large can be beneficial to mental health throughout development and may even buffer against some of the negative mental health outcomes associated with ACEs in adulthood.

Similar to Skirka [33], we also found student-athletes to score higher on hardiness than non-student-athletes. Thus, sport involvement over time may have led to the internalization of hardy attitudes (e.g., being highly committed to goal-directed behavior, seeing challenges as opportunities for growth, etc.). However, student-athletes were also less likely to experience ACEs than non-student-athletes (see Figure 1), which may have contributed to them being more likely to internalize hardy attitudes because participants with no ACEs, on average, reported greater hardiness than those with one ACE and/or multiple ACEs. Thus, it may be that sport involvement promoted hardy attitudes in student-athletes, but they may have already been more likely to develop hardy attitudes due to being less likely to experience ACEs. For individuals who reported no ACEs, it may be that there was a stronger belief in having control over events in their life due to not having as many experiences with negative events that were out of their control (e.g., maltreatment from a parent while growing up). Nevertheless, given the potential for the buffering effect athletics seems to provide to ACEs as well as the increased likelihood for developing hardy attitudes, professionals involved in working with youth (e.g., teachers, coaches, counselors) may want to consider encouraging athletic involvement for the multiple pathways it provides to promoting mental health and resilience.

### 4.2. Study Limitations

The present study is not without limitations. First, the study was a small sample from one university in the Midwest U.S., which may hinder the generalizability of the results. A larger sample size could have also allowed for grouping participants into traditional ACEs categorizations (i.e., 0, 1, 2, 3, and 4+; [1]), increasing our knowledge of ACEs’ impact on student-athletes relative to non-student-athletes in the manner researchers typically contextualize ACEs and their relationship with adult health outcomes. Additionally, only a small subset of student-athletes reported experiencing one or more ACEs, which may have impacted our ability to make comparisons between groups of individuals who have experienced ACEs. Second, we utilized a cross-sectional design, so drawing conclusions about the data is difficult. For example, it is unclear whether athletic involvement facilitated the development of hardy attitudes for participants in this study or if individuals with hardy attitudes were already more likely to self-select into staying involved with sports throughout their youth and into college. Further, it is hard to ascertain how other aspects of sport involvement (e.g., regular physical activity) impacted allostatic load for those student-athletes and non-student-athletes that experienced ACEs throughout their development. Adding to this, we only know whether or not our participants were playing sports in college, but it may be possible that some participants were active in sports for most of their lives but ceased playing once they entered their college years (thus, they may have only been out of sports for a short period of time). Third, the number of variables we collected was limited regarding the scope of mental health concerns we assessed (i.e., depressive symptoms and emotion regulation difficulties). Research collecting a more robust assessment of mental health (e.g., inclusion of anxiety measures, substance use measures, etc.) may provide greater insight into the adult psychological outcomes associated with ACEs and athletic involvement between student-athletes and non-student-athletes. Lastly, since data collection was performed for separate studies, the data collected for this study was obtained through two different sampling methods. Namely, one set of data was collected through an Introduction to Psychology research pool, wherein participants completed measures for course credit. The other set of data was collected through paying participants $5.00 for their involvement in the study. Therefore, it is unclear if these alternate forms of incentives may have differentially impacted participants’ responses and/or response style. For example, student-athletes who elected to participate via email recruitment may have been more willing to participate if they had not experienced a high degree of childhood adversity as opposed to those who completed the survey for course credit, resulting in a self-selection bias.

### 4.3. Directions for Future Research

Directions for future research may build off the limitations of this study. Research using a larger sample size involving participants from multiple universities across a wide geographic region may enhance the external validity of results. Further, a larger sample size may provide researchers the ability to create comparison groups based off the traditional ACEs groupings (i.e., 0, 1, 2, 3, and/or 4+ ACEs; [1,4]), which would provide a more comprehensive understanding of how ACEs impact student-athletes and non-student-athletes. Unfortunately, due to our sample size, we were not able to create groups of adequate size to do comparisons in this manner. To add to the generalizability of results, a longitudinal approach that collects many mental health variables and tracks participants from childhood to adulthood would provide a more nuanced understanding of how ACEs and athletic involvement relate to mental health across the lifespan, especially if that data is collected in the same manner for all participants to reduce bias of differing sampling methods. Finally, future researchers are encouraged to add validity checks when conducting online data collection [54]. Such checks may enhance the trustworthiness of results.

## 5. Conclusions

To the authors’ knowledge, this is the first study directly comparing ACEs and mental health correlates in adulthood with a sample of collegiate student-athletes and non-student-athletes. Results indicate that student-athletes tend to display fewer depressive symptoms than non-student-athletes. Similar to past research, we also found that the greater the exposure to ACEs, regardless of student-athlete status, the greater the risk to mental health outcomes. Furthermore, exposure to ACEs appeared to impact mental health at the same rate for both student-athletes and non-student-athletes. This research suggests the importance athletic involvement could play throughout childhood development in mitigating the mental health risks associated with ACEs. However, further research is warranted to draw more robust conclusions.

## Figures and Tables

**Table 1:** Descriptive statistics for measures of depressive symptoms, difficulties in emotion regulation, and hardiness.

Student-Athlete Status	ACEs Grouping	PHQ-9 Total			DERS Total			DRS Total		
		*M*	*SD*	*n*	*M*	*SD*	*n*	*M*	*SD*	*n*
Student-Athlete	No ACEs	5.10	4.66	68	80.48	20.33	69	30.32	5.08	72
	1 ACE	4.82	6.31	22	92.27	16.86	22	29.72	4.96	22
	Multiple ACEs	9.43	7.49	21	97.24	24.67	21	28.43	5.52	21
	Total	5.86	5.83	111	85.94	21.62	112	29.86	5.14	115
Non-Student-Athlete	No ACEs	6.30	5.30	69	84.93	21.39	69	30.14	5.32	69
	1 ACE	8.88	6.66	41	92.39	22.64	41	26.56	4.86	41
	Multiple ACEs	13.74	6.50	39	98.12	22.05	39	26.56	6.55	39
	Total	8.96	6.71	149	90.44	22.46	149	28.22	5.80	149
Total	No ACEs	5.71	5.01	137	82.70	20.91	138	30.23	5.18	141
	1 ACE	7.46	6.78	63	92.35	20.66	63	27.67	5.09	63
	Multiple ACEs	12.23	7.11	60	97.82	22.80	60	27.22	6.23	60
	Total	7.64	6.52	260	22.80	88.51	261	28.94	5.57	264

*Note:* PHQ-9 = Patient Health Questionnaire; DERS = Difficulties in Emotion Regulation Scale; DRS = Dispositional Resilience Scale.

**Table 2:** Summary of MANOVA and MANCOVA results.

	MANOVA				MANCOVA			
	Wilks’ Λ	*F*	Partial *η*^2^	1 − *β*	Wilks’ Λ	*F*	Partial *η*^2^	1 − *β*
ACEs Grouping	0.84	11.74 ***	0.08	1.00	0.87	8.98 ***	0.07	0.99
Status	0.93	9.85 ***	0.07	0.98	0.95	7.17 ***	0.05	0.93
Hardiness					0.78	34.57 ***	0.22	1.00
ACEs Grouping x Status	0.96	2.44 *	0.02	0.70	0.97	2.21	0.02	0.65

*Note*. * *p* < 0.05, *** *p* < 0.001. Status = Student-Athlete Status.

**Table 3:** Comparison of follow-up ANOVAs, ANCOVAs, and pairwise comparisons.

	ANOVAs				ANCOVAs			
	*F*		Mean Difference		*F*		Mean Difference	
	PHQ	DERS	PHQ	DERS	PHQ	DERS	PHQ	DERS
ACEs	19.89 ***	11.27 ***			15.98 ***	5.78 **		
None—One			−1.14	−9.79 *			−0.22	−6.23
None –Mult.			−5.88 ***	−15.15 ***			−4.83 ***	−10.37 **
One—Mult.			−4.74 ***	−5.35			−4.61 ***	−4.15
Status	15.53 ***	0.43			9.53 **	0.12		
SA—NSA			−3.19 ***	−1.93			−2.32 **	0.95

*Note*: * *p* < 0.05, ** *p* < 0.01, *** *p* < 0.001. Bonferroni adjustment made for multiple comparisons. PHQ = Patient Health Questionnaire. DERS = Difficulties in Emotion Regulation Scale; ACEs = ACEs Grouping; None = No ACEs; One = One ACE; Mult. = Multiple ACEs; Status = Student-Athlete Status; SA = Student-Athlete; NSA = Non-Student-Athlete.

## Data Availability

The data presented in this article are not readily available due to technical limitations. Requests to access the data should be directed to Matthew Powless at mp346@evansville.edu.

## Abbreviations

The following abbreviations are used in this manuscript:

ACEs	Adverse Childhood Experiences
PHQ-9	Patient Health Questionnaire
DERS	Difficulties in Emotion Regulation Scale
DRS-15	Dispositional Resilience Scale
MANOVA	Multiple Analysis of Variance
MANCOVA	Multiple Analysis of Covariance
ANOVA	Analysis of Variance
ANCOVA	Analysis of Covariance
Status	Student-Athlete Status
SA	Student-Athlete
NSA	Non-Student-Athlete
Mult.	Multiple
